# Multidrug-resistant *Mycobacterium tuberculosis*, Bangui, Central African Republic

**DOI:** 10.3201/eid1209.060361

**Published:** 2006-09

**Authors:** Laurent X. Nouvel, Eric Kassa-Kelembho, Tiago Dos Vultos, Germain Zandanga, Jean Rauzier, Carmen Lafoz, Carlos Martin, Jesus Blazquez, Antoine Talarmin, Brigitte Gicquel

**Affiliations:** * Institut Pasteur, Paris, France;; † Institut Pasteur, Bangui, Central African Republic;; ‡ Universidad de Zaragoza, Zaragoza, Spain;; § Centro Nacional de Biotecnología, Madrid, Spain

**Keywords:** Tuberculosis, Antibiotic resistance, Molecular epidemiology, dispatch

## Abstract

We investigated multidrug-resistant (MDR) *Mycobacterium tuberculosis* strains in Bangui, Central African Republic. We found 39.6% with the same spoligotype and synonymous single nucleotide polymorphism in the *mutT1* gene. However, strains had different *rpoB* mutations responsible for rifampin resistance. MDR strains in Bangui may emerge preferentially from a single, MDR-prone family.

Tuberculosis (TB) is a major public health problem and causes 2 million deaths each year. Ninety-five percent of cases are in developing countries, where limited healthcare resources lead to incomplete case and contact tracing, inadequate treatment, and as a consequence, to a larger drug resistance problem (*1**,**2*). Multidrug-resistant TB (MDRTB), defined as resistant to at least rifampin and isoniazid, is more difficult to treat and can cost 100× more than susceptible TB; it is associated with a high death rate in HIV-infected patients (*3**,**4*). MDRTB results from the selection of MDR strains in patients who failed to complete chemotherapy with the correct combination of drugs. The typing of MDR strains can be used to describe transmission and outbreaks, as shown by the identification of MDR epidemics due to the Beijing/W family strains (*5*). Other types, including Haarlem and *Mycobacterium bovis* isolates, have been involved in MDR outbreaks (*6**,**7*). Because MDR strains carry mutations in major metabolic pathways, some researchers have suggested that they may be less virulent and less transmissible (*8*); however, the occurrence of epidemics involving these strains would seem to contradict this suggestion. As with other MDR bacterial species, they may have emerged from strains more adapted to the local population (*9*). We have previously described variations in putative anti-mutator genes in Beijing/W isolates that may have favored adaptive mutations in this family of strains. The failure to show mutator phenotypes in Beijing/W strains suggests that this role may have been transient (*10**,**11*).

We studied MDRTB strains in Bangui, Central African Republic (CAR), because little information has been collected concerning MDRTB in sub-Saharan Africa. In CAR, the incidence of TB is estimated to be 250 per 100,000 inhabitants, and 1.1% of cases are MDR (*12*). In Bangui, ≈15% of the sexually active population is infected with HIV. We spoligotyped MDR strains collected by the Pasteur Institute of Bangui and looked for diversity in a series of putative anti-mutator genes.

## The Study

We studied 53 MDR *M. tuberculosis* strains isolated from different patients between 1993 and 2001 at the Bangui Pasteur Institute. Fourteen of these patients were HIV positive, 30 were HIV negative, and 9 were of undetermined status. Epidemiologic enquiries did not show a social link among patients (unpub. data). A non-MDR, nonbiased control group, which included 263 *M. tuberculosis* and 2 *M. bovis* strains, was also studied. These strains included all those from the cohort studied by Espinal et al. (*3*) for which a subculture was obtained.

**Figure A1:**
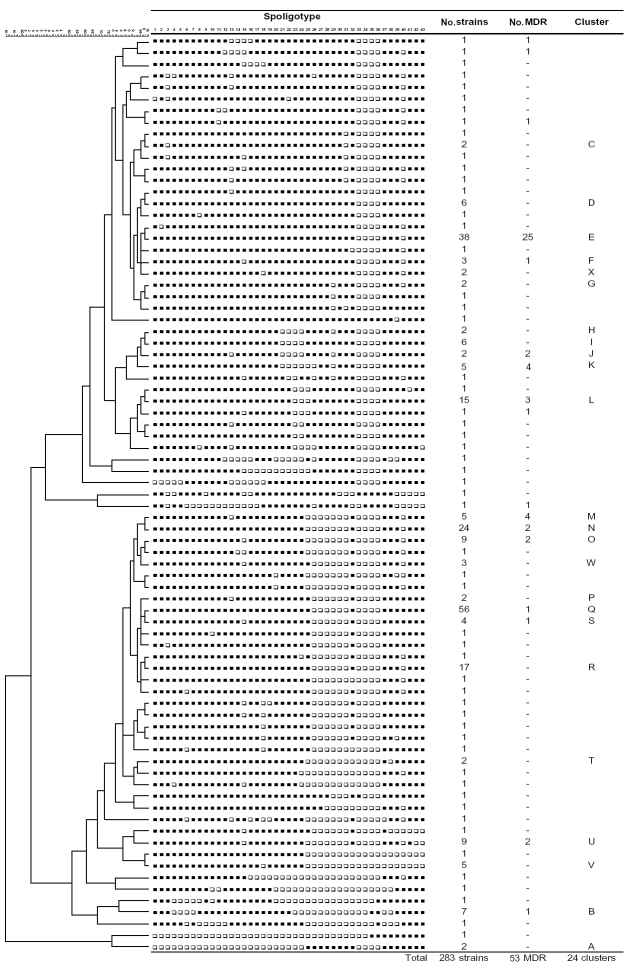
Dendrogram of patterns of the 283 strains spoligotyped (53 multidrug-resistant [MDR] and 230 non-MDR strains). For a printer friendly version of Figure A1, available at http://wwwnc.cdc.gov/eid/pdfs/06-0361-FA1.pdf.

All 318 isolates were typed by using the spoligotyping method previously described (*13*). Spoligotypes were obtained for 283 (53 MDR and 230 non-MDR strains) of the 318 isolates and were analyzed with BioNumerics software (Applied Maths, Kortrijk, Belgium). Dendrograms were constructed according to degree of similarity (Dice coefficient) and comparison with known spoligotypes. Seventy-nine different spoligotypes were identified: 55 included only a single strain, and 24 included 2–56 strains. The 24 clusters were named A to X (Figure A1).

The 53 MDR strains clustered in 8 clusters (Figure, panel A). Twenty-five (47.2%) of 53 clustered in type E, which has characteristics of the T family (ancient *M. tuberculosis* strains with numerous spacers [14]). The 230 spoligotype patterns of non-MDR strains were grouped into 22 clusters, and spoligotype E was not a major cluster (Figure, panel B). Cluster Q contained the largest number of strains. Its spoligotype is identical to the DB3 pattern ST47 characteristic of the Haarlem family (*15*). Spoligotypes 97%–99% identical with profiles characteristic of the Haarlem family of strains represent 155 strains. These observations confirm the predominance of the Haarlem type in Africa. However, the Haarlem family was not predominant in our collection of MDR isolates.

**Figure:**
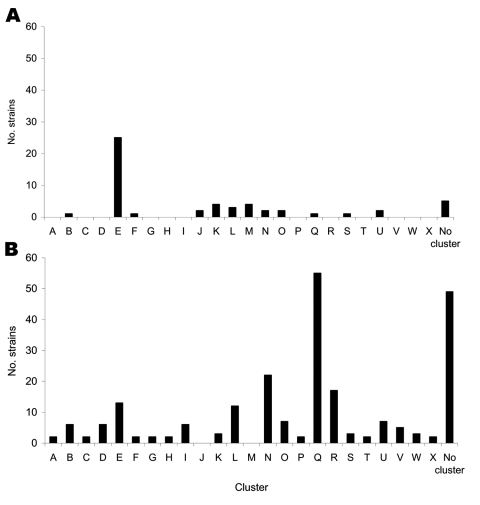
Strain distribution into various clusters observed among 53 spoligotyped multidrug-resistant (MDR) strains (A) and 230 spoligotyped non-MDR strains (B).

The clustering of MDR strains suggested an MDR outbreak; therefore, we looked for other characteristics in cluster E isolates. First, we tested for diversity in the *rpoB* region, which was likely to be responsible for rifampin resistance. Five variants were found among the 26 MDR strains that constituted clusters E and F (Table): 9 had a Ser-531 (TCG) to Leu (TTG) substitution; 8 and 5 strains contained a substitution of His-526 (CAC) with Tyr (TAC) and Arg (CGC), respectively; 3 had an Asp-516 (GAC) to Val (GTC) variant; and 1 a Leu-533 (CTG) to Pro (CCG) substitution. All these variations are in the rifampin resistance-determining region frequently encountered in strains with a rifampin-resistant phenotype (*8*). These variants probably determine rifampin resistance and may have occurred independently, not necessarily corresponding to MDRTB transmission, even for strains of the same cluster with the same change in *rpoB*. Indeed, sociodemographic and epidemiologic characterization of the patients did not show any links between these MDRTB cases. Therefore, rifampin resistance seems to have been acquired independently and repeatedly by cluster E and F strains. To find a way to reduce the dissemination of such strains likely to generate MDR isolates, we characterized strains of cluster E and F. In particular, we looked for single nucleotide polymorphisms (SNPs) in the putative genes *mutT1*, *mutT2*, *mutT3*, *Rv3908*, *mutY*, *mutM*, *ada*/*alkA*, and *ogt*. Sequencing was performed as previously described (*11*). With reference to published *M. tuberculosis* sequences, we found 1 synonymous SNP in *mutT1* corresponding to Val 265 (GTC) to Val (GTA). This SNP is only present in strains of cluster E and strains 27 (cluster F), 28, and 29 and is absent from all strains in other clusters. Therefore, these MDR strains are characterized by a spoligotype pattern (ST 52 ± spacer 11 or 12 to 15 and ST 107) and the presence of the *mutT1* SNP 265.

**Table:** *rpoB* mutations observed in strains of cluster E and F*

Cluster	No.	ATB	*rpoB*	*mutT1*
E	7	R/I/E	Asp GAC 516 Val GTC	Val GTC 265 Val GTA
E	8	R/I/S	Asp GAC 516 Val GTC	Val GTC 265 Val GTA
E	9	R/I/E/S	Asp GAC 516 Val GTC	Val GTC 265 Val GTA
E	10	R/I/S	His CAC 526 Arg CGC	Val GTC 265 Val GTA
E	11	R/I/E/S	His CAC 526 Arg CGC	Val GTC 265 Val GTA
E	45	R/I/E/S	His CAC 526 Arg CGC	Val GTC 265 Val GTA
E	46	R/I/E/S	His CAC 526 Arg CGC	ND
E	12	R/I/E/S	His CAC 526 Asp GAC	Val GTC 265 Val GTA
E	13	R/I/E	His CAC 526 Tyr TAC	Val GTC 265 Val GTA
E	14	R/I/E/S	His CAC 526 Tyr TAC	Val GTC 265 Val GTA
E	15	R/I/E	His CAC 526 Tyr TAC	Val GTC 265 Val GTA
E	16	R/I/S	His CAC 526 Tyr TAC	Val GTC 265 Val GTA
E	17	R/I/E/S	His CAC 526 Tyr TAC	Val GTC 265 Val GTA
E	18	R/I	His CAC 526 Tyr TAC	Val GTC 265 Val GTA
E	19	R/I/E	His CAC 526 Tyr TAC	Val GTC 265 Val GTA
E	47	R/I/E/S	His CAC 526 Tyr TAC	ND
E	20	R/I/E	Ser TCG 531 Leu TTG	Val GTC 265 Val GTA
E	21	R/I/E/S	Ser TCG 531 Leu TTG	Val GTC 265 Val GTA
E	22	R/I/E/S	Ser TCG 531 Leu TTG	Val GTC 265 Val GTA
E	23	R/I/E/S	Ser TCG 531 Leu TTG	Val GTC 265 Val GTA
E	24	R/I/E	Ser TCG 531 Leu TTG	Val GTC 265 Val GTA
E	44	R/I/E/S	Ser TCG 531 Leu TTG	Val GTC 265 Val GTA
F	27	R/I/S	Ser TCG 531 Leu TTG	Val GTC 265 Val GTA
E	49	R/I/E/S	Ser TCG 531 Leu TTG	Val GTC 265 Val GTA
E	50	R/I/E/S	Ser TCG 531 Leu TTG	ND
E	48	R/I	Leu CTG 533 Pro CCG	ND

*ATB, antibiogram results, indicates strains resistant to rifampin (R), isoniazid (I), ethambutol (E), and streptomycin (S); ND, not determined.

MDR strains of clusters E and F and strains 28 and 29 (a single difference in spacers between E and F, strain 28 or 29) corresponded to 9 new cases and 19 patients who had received previous treatment. MDR strains in other clusters corresponded to 9 new cases, 15 previously treated patients, and 1 case for which no history was available (*3*).

## Conclusions

We used 2 types of markers to study the genetic diversity of MDR *M. tuberculosis* strains isolated in Bangui: spoligotyping and SNPs in a series of putative DNA repair genes. Many MDR strains were clustered in 1 spoligotype and carried the same SNP in the anti-mutator gene *mutT1*. Indeed, 25 of the 53 MDR strains were in cluster E. Thirty-two percent of these MDR strains were from new cases of infection, and 40% were from HIV-infected patients. This cluster was not a major cluster among the 265 non-MDR isolates collected during a 5-month period. The same SNP was found in all strains of cluster E and F tested and in 2 strains that differed by 1 spacer. These strains carry variants of *rpoB* that confer rifampin resistance, which implies that these strains do not correspond to an MDR-TB outbreak. However, this finding is consistent with the possibility that these strains represent an MDR-prone family, members of which are often associated with MDR phenotypes in Bangui. Detection of strains characterized by the T family spoligotype and *mutT1* SNP 265 may be useful to identify patients at risk of developing MDR-TB.
